# Implementation of shared decision-making about rooming-in: A before and after an audit of breastfeeding in Taiwan

**DOI:** 10.1186/s13006-024-00649-6

**Published:** 2024-06-04

**Authors:** Hsiao-Ying Hung, Chun-Che Wen, Pei-Fang Su, Shek-Yip Man, Ying-Ju Chang

**Affiliations:** 1 Department of Nursing, College of Medicine, National Cheng Kung University, Tainan, Taiwan; 2 Department of Nursing, National Cheng Kung University Hospital, Tainan, Taiwan; 3 Department of Public Health Sciences, Medical University of South Carolina, Charleston, USA; 4 Department of Statistics, National Cheng Kung University, Tainan, Taiwan; 5 Institute of Allied Health Sciences, College of Medicine, National Cheng Kung University, Tainan, Taiwan

**Keywords:** Shared decision-making, Prenatal intention, Rooming-in, Breastfeeding, Maternal autonomy

## Abstract

**Background:**

The 24-h rooming-in policy is crucial to the Baby-Friendly Hospital Initiative (BFHI) for promoting breastfeeding. However, this policy may restrict maternal autonomy. In 2018, to integrate women’s preferences into care decisions, Taiwan’s Baby-Friendly certification included prenatal shared decision-making (SDM) for rooming-in. Prior to 2018, maternal knowledge, considerations, and intentions regarding rooming-in and the impact of prenatal SDM were unknown.

**Methods:**

A retrospective electronic medical record cohort study was conducted in southern Taiwan. Data on healthy postpartum women eligible for rooming-in and breastfeeding for the years 2017 and 2019, reflecting the periods before and after prenatal SDM was introduced, were gathered. Maternal and newborn characteristics, maternal knowledge, considerations, and prenatal intentions for postpartum rooming-in and breastfeeding during hospitalization were collected. Additionally, data on actual postpartum rooming-in practices during hospitalization and exclusive breastfeeding (EBF) practices from birth to hospital discharge, to 1 month, and to 2 months postpartum were collected. Descriptive and non-parametric statistics were applied to analyze the data.

**Results:**

A total of 621 women in 2017 and 311 women in 2019 were included. After prenatal SDM was introduced, the rooming-in rate during hospitalization fell from 42.2% in 2017 to 25.6% in 2019 (*p* < 0.001), and the EBF rate declined from 45.9% to 35.7% (*p* = 0.01). Additionally, the 1-month postpartum EBF rate decreased from 46.4% in 2017 (*n* = 571) to 44.3% in 2019 (*n* = 264), and the 2-month postpartum EBF rate dropped from 45.5% in 2017 (*n* = 591) to 40.2% (*n* = 308). According to the 2019 Patient Decision Aids responses (*n* = 236), women demonstrated limited understanding of rooming-in, with only 40.7% expressing an intention toward 24-h rooming-in. Women of older maternal age (*p* < 0.001), with a graduate degree (*p* = 0.02), full-time employment (*p* = 0.04), and concerns about rest disruption (*p* < 0.001), were more likely to prefer non-24-h rooming-in.

**Conclusions:**

Initiatives must promote prenatal SDM to enable healthcare providers to address misconceptions and tailor education, thereby increasing women’s intention toward 24-h rooming-in and EBF. Future research should explore women’s experiences and unmet needs at BFHI facilities to inform the construction of a baby- and mother-friendly environment.

## Background

The Baby-Friendly Hospital Initiative (BFHI) was launched by the World Health Organization (WHO) and the United Nations International Children’s Emergency Fund in 1991 to support and promote breastfeeding [1]. With a concerted global effort, the WHO reported that in 14% of countries with more than 50% of births occurring in BFHI facilities, 47% of newborns started breastfeeding within one hour of being born, and 48% of infants under six months of age were exclusively breastfed [2].

In response to Taiwan’s low exclusive breastfeeding (EBF) rate of only 5.4% during the 1970s, the government adopted the WHO’s BFHI in 2000 to promote breastfeeding [3]. BFHI was significantly promoted, increasing the number of accredited institutions in Taiwan from 38 in 2001 to 163 in 2021, accounting for 73.4% of births [4]. The Joint Commission of Taiwan, responsible for BFHI accreditation, reported that the EBF rate during hospitalization rose from 29.4% in 2001 to 48.4% in 2006. However, after 2006, the annual decline in the rate of EBF during hospitalization was considerable, falling to 33.4% by 2018 [5]. Regrettably, the EBF rate for infants under six months of age in Taiwan—defined as infants receiving only mothers’ milk, with no other foods or liquids from birth until six months [6]—has also shown a declining trend. According to the Taiwan Health Promotion Administration, the rate fell from 48.7% in 2013 to 37.9% in 2021 [4, 7]. This decrease underscores the urgent need to reassess breastfeeding advocacy strategies.

Endorsement of the BFHI policy for breastfeeding is not without controversy. Promoting breastfeeding as the only appropriate option for feeding infants presents a moral problem for some women [8]. Recommendations from international organizations, along with medical and nursing associations, suggest that all mothers should breastfeed unless compelling reasons advise otherwise. When a mother chooses not to breastfeed, even for a justifiable reason, she could be viewed as inadequate, taking unnecessary risks, and acting unethically [9, 10]. Such a portrayal may evoke feelings of frustration, shame, and guilt, adversely affecting the well-being of vulnerable new mothers. Such feelings could lead mothers to doubt and even reject healthcare providers’ recommendations regarding infant feeding [11, 12]. This skepticism may arouse women’s desire for autonomy and accountability for their own health and their babies’ health [13]. Hence, women’s perspectives, experiences, and preferences must be carefully incorporated into care for both the mother and the newborn.

Shared decision-making (SDM) is an interactive process of communication in which patients and clinicians collaborate to reach optimal care decisions aligned with patients’ preferences. The urgency of implementing SDM in perinatal care is highlighted by the 2021 guidelines from the United Kingdom’s National Institute for Health and Care Excellence and the WHO’s recommendations, both of which emphasize respecting a woman’s autonomy in making her own care choices [14, 15]. Despite this emphasis, most research on women’s experiences with SDM in maternity care has been conducted in Europe, the Americas, or Australia [13]. Studies on maternity care SDM in Asia are limited, with the majority focusing on birth choices [16, 17].

Given the widespread promotion of the importance of women’s autonomy in perinatal care, women’s organizations and maternal experts in Taiwan have urged government policymakers and the public to prioritize the rights and interests of women during the peripartum period. Additionally, with the abundance of relevant digital media information in Taiwan, women have become more knowledgeable and are seeking information beyond what healthcare providers offer. Although such increased autonomy is often beneficial, it may also have unintended consequences. For example, Step 7 of the BFHI states, “Enable mothers and their infants to remain together and to practice rooming-in 24 h a day,” which underlines the benefits of keeping newborns with their mothers for the first 24 h after birth. This practice fosters mother–infant interaction, supports feeding in accordance with the demands of the newborn, stimulates breast milk production, and encourages breastfeeding [18, 19]. However, 24-h rooming-in was mandatory for Taiwan’s BFHI accreditation prior to 2018, leading some postpartum women to report overwhelming exhaustion due to the lack of prompt nursing assistance and individualized care [20–22]. As a result, despite the benefits of rooming-in for both mothers and children, there was an increased demand for more autonomy and alternative care options for their newborns.

In response to mothers’ preferences, the Joint Commission of Taiwan amended the 24-h rooming-in requirement from a “mandatory” criterion to an “optional” criterion for BFHI accreditation in 2018. The Joint Commission further stressed that decisions regarding postpartum rooming-in and breastfeeding should be made in prenatal SDM [23]. During prenatal SDM sessions, healthcare providers should inform pregnant women and their families about the various rooming-in options available to ensure a comprehensive understanding of the pros and cons of each option. They should then work with the women and their families to make the optimal decision for postpartum newborn care [24, 25]. To support implementation of prenatal SDM, the Joint Commission introduced Patient Decision Aids (PDAs) to assist healthcare providers in assessing women’s knowledge of, and considerations regarding, rooming-in and breastfeeding and identifying possible misunderstandings that may impede optimal decision-making [26, 27].

Although Taiwan promoted the BFHI for several years, SDM regarding rooming-in and breastfeeding was not introduced until 2018. Consequently, women’s intentions to room in and breastfeed, and their knowledge of and considerations regarding these practices, were not well explored before 2018. The adoption of prenatal SDM has prompted several concerns among BFHI institutions regarding its potential influence on postpartum rooming-in and breastfeeding practices. Thus, this study (1) examined Taiwanese women’s intentions, knowledge, and considerations regarding rooming-in and breastfeeding during their hospitalization and (2) assessed the influence of prenatal SDM on postpartum rooming-in and breastfeeding practices.

## Methods

### Design

A retrospective cohort study was conducted at a BFHI-accredited medical center in southern Taiwan.

### Participants

After approval to conduct the study was granted by the Institutional Review Board (No. A-ER-109–185), the electronic medical records (EMRs) of two groups of healthy postpartum women aged 18 years or older were analyzed. These women had no postpartum complications, contraindicating rooming-in or breastfeeding, and delivered healthy full-term neonates between January and December 2017 and between January and December 2019. As prenatal decision-making was influenced by infection control policies during the COVID-19 pandemic from 2020 to 2023, records were limited to 2019, prior to the onset of the pandemic. Sample sizes were not estimated prior to conducting the medical review to maximize the sample size and accommodate the cohort study design.

### Data collection

Data from the EMR were extracted for maternal and neonatal characteristics of interest. The data included (1) maternal demographic traits such as age, education level, marital status, and employment status; (2) maternal obstetric characteristics, including parity, number of fetuses, and mode of delivery; (3) neonatal characteristics, such as gestational age at birth (weeks) and birth weight (grams); (4) maternal knowledge, considerations, and prenatal intentions regarding postpartum rooming-in and breastfeeding during hospitalization; (5) actual postpartum rooming-in practices during hospitalization; and (6) actual breastfeeding practices from birth to hospital discharge, to 1 month, and to 2 months postpartum.

The EMRs included PDAs documenting women’s knowledge, considerations, and intentions regarding postpartum rooming-in and breastfeeding during hospitalization. The PDAs were developed by the Joint Commission of Taiwan using expert consensus [27]. The PDAs responses were only available for the 2019 cohort of postpartum women.

The PDAs for rooming-in include three subscales: knowledge, considerations, and intention. The knowledge subscale includes five questions, with each question rated as “yes” or “no.” Each correct response earns 1 point, resulting in a maximum score of 5. The considerations subscale comprises seven items, with endpoints ranging from 1 (*least important/concerning*) to 5 (*most important/concerning*). The single item for prenatal intention for rooming-in offers 3 options: 24-h rooming-in, partial rooming-in, and separate care [25, 27].

The PDAs for breastfeeding include three subscales: knowledge, considerations, and intention. The knowledge subscales include six questions, with each question rated as “yes” or “no.” Each correct response earns 1 point, resulting in a maximum score of 6. The considerations subscale comprises six items, with endpoints ranging from 1 *(least important)* to 5 *(most important)*. The single item for prenatal intentions of breastfeeding offers three options: exclusive breastfeeding, mixed feeding, and formula feeding [27]. Exclusive breastfeeding was defined as infants who only received breast milk without any additional liquids or solids, including water. Mixed feeding was defined as being fed a combination of breast milk and other liquids. Formula feeding was defined as feeding with artificial formula.

During the postpartum hospital stay, each woman’s rooming-in and breastfeeding practices were documented based on their actual status. Rooming-in could be recorded as either 24-h rooming-in, partial rooming-in, or separate care, and breastfeeding could be recorded as exclusive breastfeeding, mixed feeding, or formula feeding. After discharge, breastfeeding practices from birth to 1 month and to 2 months postpartum were documented through structured follow-up phone calls. Before posing specific questions, nurses ensured that mothers understood the definitions of EBF, mixed feeding, and formula feeding, emphasizing that EBF involves feeding the baby only breast milk without any supplemental liquids or solids, including water. At one month postpartum, the question posed was “How has your baby been fed in the first month of life?” At two months postpartum, the question was, “How has your baby been fed in the first two months of life?” [28–30].

### Data analysis

Continuous variables are presented as the means and standard deviations (SDs), while categorical variables are presented as percentages. For the analysis of continuous variables, the Wilcoxon rank sum test was applied to compare characteristics, postpartum rooming-in during hospitalization, and breastfeeding practices from birth to hospital discharge, to 1 month, and to 2 months postpartum for the 2017 and 2019 cohorts. The analysis of categorical variables involved the use of chi-square tests, and Fisher’s exact test was utilized when any expected cell size was less than 5. Kruskal–Wallis tests were conducted to examine factors influencing women’s prenatal rooming-in decisions. If the Kruskal–Wallis test indicated a significant difference, Dunn’s test was performed as a post hoc analysis, applying a Bonferroni correction with α set at 0.017. Non-parametric statistics were applied due to the non-normal distribution of the data.

All analyses were performed using SAS version 9.4 (SAS Institute) and R version 4.1.1 (R Foundation for Statistical Computing, Vienna, Austria), with the significance threshold set at α = 0.05.

## Results

### Characteristics of the participants

Table 1 displays the characteristics of the cohorts from 2017 and 2019, with 621 women in 2017 and 311 in 2019. The average maternal age was 32.7 years (range 18-49) in 2017 and 33.0 years (range 18-45) in 2019. The proportion of women aged 35 or older was 38.3% in 2017 and 36.0% in 2019. Most of the women were married and had a bachelor’s degree. Nearly half were employed full-time. Of these, approximately half were first-time mothers, most of whom were carrying a single birth and undergoing VD. The average gestational age at delivery was approximately 39 weeks, with newborns averaging 3,200 grams. Although a greater share of the women were first-time mothers in 2017 (51.4%) than in 2019 (43.7%), no significant differences in other demographic or obstetric characteristics were detected between the two cohorts

**Table 1:** Characteristics of 2017 and 2019 cohorts (*N* = 932)

	2017 (*n* = 621)	2019 (*n* = 311)	*p*-value^+^
**Maternal Age** (range)	32.7 ± 4.6 (18–49)	33.0 ± 4.8 (18–45)	0.41
≥ 35 years olde	238 (38.3)	112 (36.0)	0.54
**Education levels**			0.07
High school or lower	91 (15.4)	50 (16.1)	
Bachelor’s degree	406 (68.6)	192 (61.9)	
Master’s degree/Doctorate	95 (16.0)	68 (22.0)	
**Marital status**			0.55
Unmarried	23 (3.7)	14 (4.5)	
Married	597 (96.3)	296 (95.5)	
**Employment**			0.12
Full-time job	259 (41.8)	146 (47.1)	
Part-time job	361 (58.2)	164 (52.9)	
**Primipara**			0.03
Yes	319 (51.4)	136 (43.7)	
**Single fetuses**	611(98.4)	304 (97.7)	0.60
**Mode of delivery**			0.07
VD	353 (56.8)	157 (50.5)	
C/S	268 (43.2)	154 (49.5)	
**Infant gestational age at birth** (weeks)	38.7 ± 0.94	39.0 ± 0.94	0.37
**Infant birth weight** (grams)	3131.4 ± 335	3162.6 ± 270	0.11

Data are presented as numbers with percentages (%), except for age, infant gestation age at birth, and infant birth weight, which are presented as mean ± standard deviation; VD = vaginal delivery. C/S = cesarean section

^+^The Wilcoxon rank sum test was used to test continuous variables; the Pearson chi-squared test was used to test categorical variables; Fisher’s exact test was used for categorical variables with cell sizes ≤ 5

**Fig. 1:**
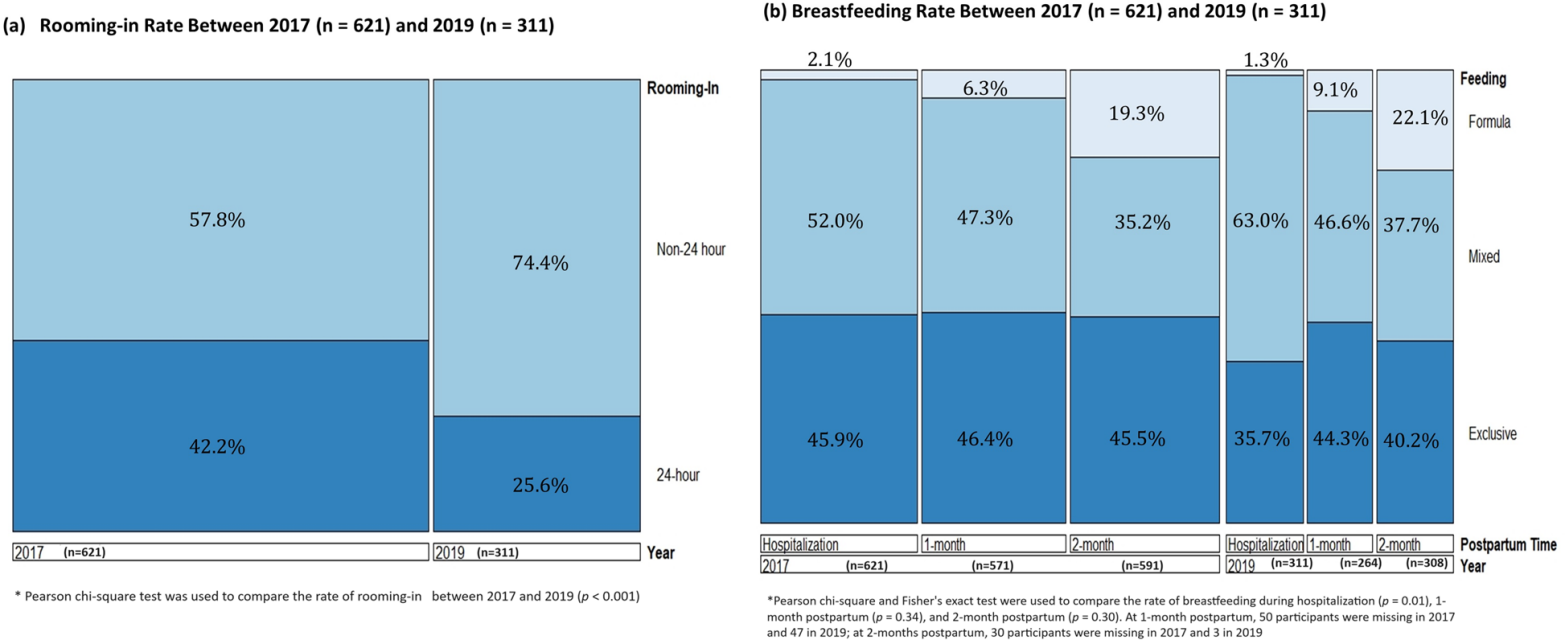
Comparison of rooming-In and breastfeeding rates between 2017 and 2019 cohorts. This figure compares the rates of rooming-in during hospitalization and exclusive breastfeeding from birth to hospital discharge, at 1 month, and at 2 months postpartum for the 2017 (*n* = 621) and 2019 (*n* = 311) cohorts.

Figure 1 indicates that women in the 2017 cohort (*n* = 621) were more likely than those in the 2019 cohort (*n* = 311) to practice 24-h rooming-in (42.2% vs. 25.6%, *p* < 0.001) and EBF (45.9% vs. 35.7%, *p* = 0.01) during their hospital stay, suggesting that the introduction of prenatal SDM was associated with lower rates of 24-h rooming-in and EBF during hospitalization. Furthermore, the rate of EBF at 1 month postpartum fell from 46.4% in 2017 (*n* = 571) to 44.3% in 2019 (*n* = 264). At 2 months postpartum, the rate declined from 45.5% in 2017 (*n* = 591) to 40.2% in 2019 (*n* = 308). Follow-up was lost in both 2017 and 2019 due to unsuccessful phone contact. In 2017, 50 participants were lost at 1 month postpartum and 30 at 2 months postpartum. In 2019, 47 participants were lost at 1 month postpartum and 3 at 2 months postpartum. Despite these apparent declines, there was no evidence of a difference in these rates due to the small sample size.

### Knowledge, considerations, and prenatal intention for breastfeeding and rooming-in

Only the responses of the 236 women who completed the PDAs questions during prenatal SDM in 2019 were included in this analysis. Table 2 shows that, prior to receiving prenatal education, the accuracy rates for rooming-in knowledge among these women varied from 53.8% to 86.4%. The accuracy rates for questions 1 to 3 were above 70%, whereas those for questions 4 and 5 were 58.5% and 53.8%, respectively. This disparity underscores women’s uncertainty about whether separate care during hospitalization increases the risk of infection for their baby and causes discomfort.

**Table 2:** Women’s prenatal knowledge of rooming-in and breastfeeding (*n* = 236)

**Rooming-in Knowledge**	**Number**	**Correct** **n (%)**
Q1: Rooming-in helps babies learn to breastfeed sooner.		204 (86.4)
Yes	204	
No	32	
Q2: During rooming-in, my baby will sleep alongside me in the same bed.		175 (74.2)
Yes	61	
No	175	
Q3: Rooming-in increases my rest time.		167 (70.8)
Yes	69	
No	167	
Q4: Separate care may increase the baby’s risk of infection.		138 (58.5)
Yes	138	
No	98	
Q5: Separate care may lead to more walking for me, potentially causing pain or other discomfort.		127 (53.8)
Yes	127	
No	109	
**Breastfeeding Knowledge**	**Number**	**Correct** **n (%)**
Q1: Breast milk reduces the risk of many acute and chronic diseases in babies.		206 (87.3)
Yes	206	
No	30	
Q2: Breastfeeding lowers my risk of developing breast cancer.		178 (75.4)
Yes	178	
No	58	
Q3: Breastfeeding helps prevent postpartum hemorrhage.		166 (70.3)
Yes	166	
No		
Q4: In the initial stages of breastfeeding, the support of professionals and family may be essential.		227 (96.2)
Yes	227	
No	9	
Q5: To avoid overfeeding, feeding amounts should be regulated in accordance with the baby’s cues.		201 (85.2)
Yes	201	
No	35	
Q6: Mixed feeding does not affect the production of breastmilk.		80 (33.9)
Yes	156	
sNo	80	

On the rooming-in subscale, the correct response for question 1, 4, and 5 is “yes,” and the correct response for question 2 and 3 is “no”. On the breastfeeding subscale, the correct response for question 1–5 is “yes,” and the correct response for question 6 is “no.”

The accuracy rates for breastfeeding knowledge ranged from 33.9% to 96.2%. The highest accuracy rate was for questions 4, which states, “In the initial stages of breastfeeding, the support of professionals and family may be essential.” The accuracy rates for questions 1 and 5 exceeded 85%, whereas those for questions 2 and 3 were approximately 70%. However, for question 6, only 33.9% of the women recognized that mixed feeding could affect breast milk production. These results emphasize the need for thorough discussion with pregnant women about the advantages of breastfeeding, such as lowering the risk of breast cancer and preventing postpartum hemorrhage, in addition to the effects of mixed feeding on breast milk production.

Table 3 presents the three primary considerations the women had about rooming-in: (1) having adequate rest, (2) worrying that rooming-in may cause discomfort, and (3) needing to quickly learn the skills necessary for newborn care. Additionally, only 40.7% of the women intended to practice 24-h rooming-in, with approximately 44.9% stating that they favored separate care during prenatal SDM.

**Table 3:** Women’s prenatal considerations and intention for rooming-in during hospitalization (*n* = 236)

**Rooming-in considerations**	**Mean**	**SD**
Q1: I will be able to breastfeed my baby at any time	3.79	1.19
Q2: I will be able to nurse my baby at any time	3.82	1.14
Q3: I will be able to quickly acquire the knowledge and skills required to care for my baby	4.14	1.04
Q4: My family will have more time and opportunities to interact with my baby if I rooming-in	3.72	1.24
Q5: I will have sufficient time for rest	4.47	0.83
Q6: Rooming-in may cause me discomfort	4.15	1.02
Q7: Rooming-in disrupts my or my roommate’s rest	3.91	1.09
**Prenatal rooming-in intention during hospitalization**	**Number**	**%**
24-h rooming-in, yes	96	40.7
Partial rooming-in, yes	34	14.4
Separate care, yes	106	44.9

The 24-h option indicates that a woman intends to stay with their baby in the same room for the entire day. The partial option indicates a woman intends to stay with their baby in the same room for only part of the day. The separate option indicates that a woman intends to have their baby cared for in a nursery room.

Table 4 presents the three primary considerations the women had about breastfeeding: (1) whether it benefits babies' health, (2) whether it offers complete nutrition for their babies, and (3) whether it benefits their own health. Notably, only 40.7% of the women stated that they intended to practice EBF during prenatal SDM.

**Table 4:** Women’s prenatal considerations, and intention for breastfeeding (*n* = 236)

**Breastfeeding considerations**	**Mean**	**SD**
Q1: Breast milk offers complete nutrition for babies	4.81	0.44
Q2: Breastfeeding benefits babies’ health	4.87	0.35
Q3: Breastfeeding benefits my health	4.69	0.60
Q4: Family opinions on how I feed my baby	3.30	1.40
Q5: Workplace support for my baby feeding choices	3.10	1.70
Q6: Financial concerns	3.20	1.50
**Prenatal breastfeeding intention during hospitalization**	**Number**	**%**
Exclusive breastfeeding, yes	96	40.7
Mixed feeding, yes	89	37.7
Formula feeding, yes	49	20.8

The exclusive breastfeeding option indicates that a woman intends to feed their infant only breast milk, without any other liquids or solids, not even water. The mixed feeding option indicates that a woman intends to feed their infant both breast milk and other forms of nutrition, such as formula. The formula feeding option indicates that a woman intends to feed their infant manufactured formula only.

### Factors influencing the prenatal intention toward rooming-in

 Table 5 presents the factors affecting women’s preferences for rooming-in. The results revealed that the women who were more likely to choose partial rooming-in or separate care were older (*p* < 0.001), had a higher education level (*p* = 0.02), and were in employed full-time (*p* = 0.04).

**Table 5:** Characteristics, considerations, and breastfeeding intention in women with differing prenatal rooming-in intention (*n* = 236)

	Prenatal SDM for rooming-in			***p***-value^**+**^	post-hoc test
	(1) 24-hour option (***n*** = 96)	(2) Partial option (***n*** = 34)	(3) Separate option (***n*** = 106)		
**Maternal Age** (years)	31.7 ± 5.2	34.1±4.7	34.0±4.1	< 0.001	(1) <(2), (1)<(3)
**Education levels**				0.02	
High school or lower	20 (20.8)	5 (14.7)	12 (11.4)		
Bachelor’s degree	65 (67.7)	19 (55.9)	63 (60.0)		
Master’s or doctorate	11 (11.5)	10 (29.4)	30 (28.6)		
**Employment**				0.04	
Full-time job	39 (40.6)	22 (64.7)	54 (50.9)		
Part-time job/unemployed	57 (59.4)	12 (35.3)	52 (49.1)		
**Primipara**				0.10	
Yes	48 (50.0)	18 (52.9)	39 (36.8)		
**Number of fetuses**				0.65	
One	95 (99.0)	33 (97.1)	104 (98.1)		
**Rooming-in considerations**					
Q1: I will be able to breastfeed my baby at any time.	4.4 ± 0.9	3.9 ±0.8	3.2 ± 1.3	< 0.001	(1) >(2), (1)>(3), (2)>(3)
Q2: I will be able to nurse my baby at any time.	4.4 ± 0.9	3.9 ±0.9	3.3 ± 1.3	< 0.001	(1)>(2), (1)>(3), (2)>(3)
Q3: I will be able to quickly acquire the knowledge and skills required to care for my baby.	4.5 ± 0.9	3.9 ±1.1	3.9 ± 1.1	< 0.001	(1)>(2), (1)>(3)
Q4: My family will have more time and opportunities to interact with my baby if I rooming-in.	4.3 ± 1.0	3.6 ±1.0	3.2 ± 1.2	< 0.001	(1)>(2), (1)>(3)
Q5: I will have sufficient time for rest.	4.3 ± 1.0	4.6 ± 0.5	4.6 ± 0.6	0.07	
Q6: Rooming-in may cause me discomfort.	4.0 ± 1.2	4.4 ± 0.8	4.3 ± 0.9	0.16	
Q7: Rooming-in disrupts my or my roommate’s rest.	3.6 ± 1.2	3.9 ± 1.0	4.2 ± 0.9	< 0.001	(3)>(1)
**Breastfeeding considerations**					
Q1: Breast milk offers complete nutrition for babies.	4.9 ± 0.3	4.8 ± 0.5	4.7 ± 0.5	0.004	(1)>(3)
Q2: Breastfeeding benefits babies’ health.	4.9 ± 0.3	4.8 ± 0.4	4.8 ± 0.4	0.083	
Q3: Breastfeeding benefits my health.	4.7 ± 0.5	4.6 ± 0.7	4.7 ± 0.6	0.55	
Q4: Family opinions on how I feed my baby	3.5 ± 1.5	3.2 ± 1.2	3.17 ± 1.4	0.20	
Q5: Workplace support for my baby feeding choices	3.4 ± 1.8	2.9 ± 1.7	3.0 ± 1.7	0.04	(1)>(2), (1)>(3)
Q6: Financial concerns	3.3 ± 1.6	2.9 ± 1.4	3.2 ± 1.4	0.07	
**Prenatal Breastfeeding Intention (** ***n*** **, %)**					
Exclusive breastfeeding	74 (77.1)	16 (48.5)	32 (30.5)	< 0.001	
Mixed/formula feeding	22 (22.9)	17 (51.5)	73 (69.5)		

The 24-h option indicates that a woman intends to stay with their baby in the same room for the entire day. The partial option indicates that a woman intends to stay with their baby in the same room for only part of the day. The separate option indicates that a woman intends to have their baby cared for in a nursery room

^+^*p*-value for statistical tests of associations between groups. The Kruskal–Wallis test was used to test continuous variables, the Pearson chi-squared test was used to test categorical variables, and Fisher’s exact test was used for categorical variables with cell sizes ≤ 5. Dunn’s test was used to test multiple comparisons after the Kruskal–Wallis test determined significance

Moreover, women who preferred partial rooming-in or separate care placed less importance on the ability to breastfeed (*p* < 0.001) or to nurse at any time (*p* < 0.001), on quickly acquiring newborn care skills (*p* < 0.001), and on opportunities for their families to interact with the baby (*p* < 0.001) than those who preferred 24-h rooming-in. As for breastfeeding, these women were less concerned with breast milk providing complete nourishment for the baby (*p* = 0.004) and workplace support for breastfeeding (*p* = 0.04). However, they had greater concerns about rooming in interrupting their own or their roommates’ rest (*p* < 0.001). Compared to those who chose 24-h rooming-in, their EBP intentions were lower.

**Fig. 2:**
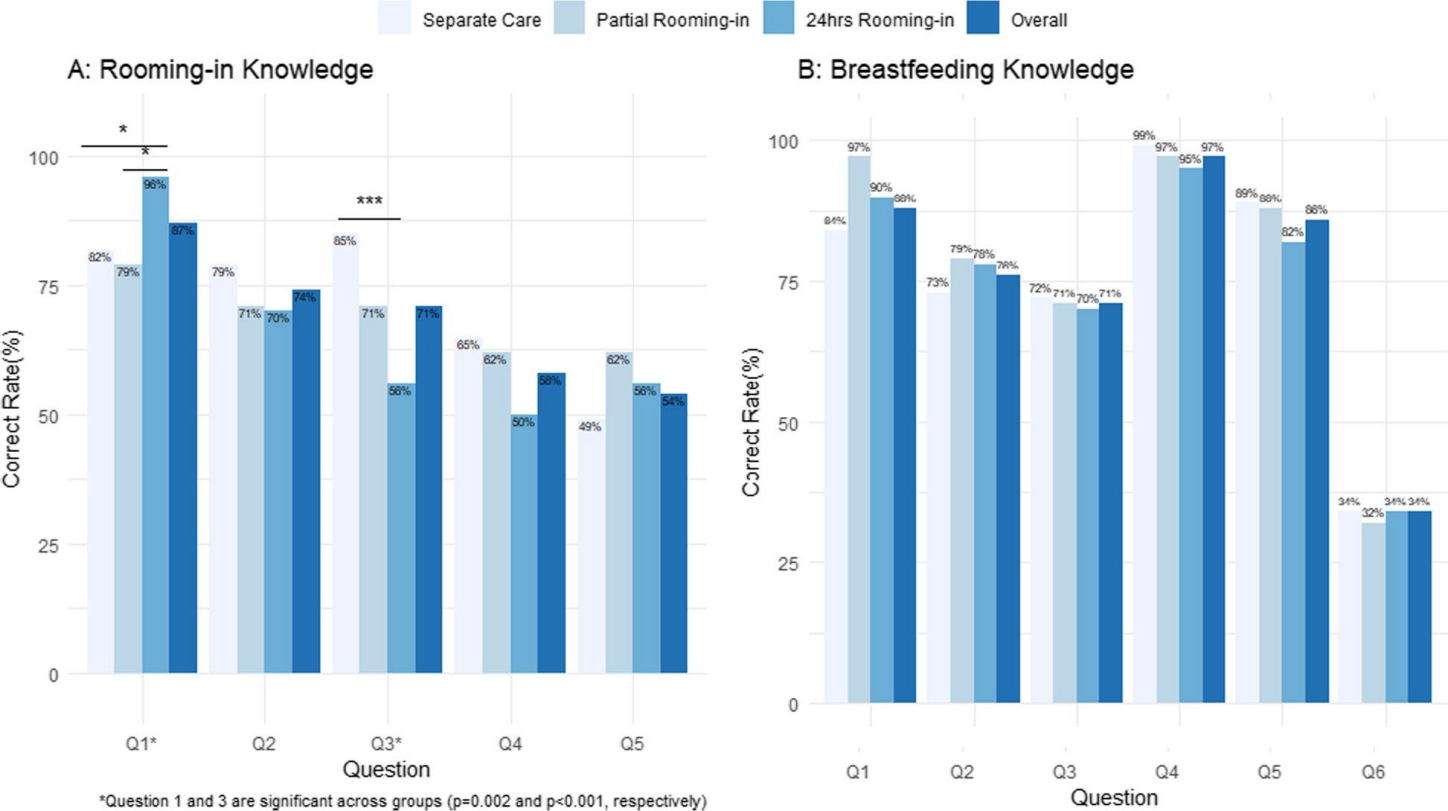
Comparison of knowledge regarding rooming-in and breastfeeding among women with differing prenatal rooming-in intentions

Figure 2 illustrates disparities in rooming-in knowledge between women with different preferences. Specifically, women who preferred partial rooming-in or separate care demonstrated better understanding of the potential for rooming-in to decrease their rest, showing higher accuracy on question 3 (“Rooming-in increases my rest time”; *p* < 0.001) compared to other groups. However, they were uncertain about the benefits of rooming-in for initiating breastfeeding, as indicated by their lower accuracy on question 1 (“Rooming-in helps babies learn to breastfeed sooner”; *p* = 0.002). No other notable differences in breastfeeding knowledge were detected between the groups.

## Discussion

This study explored women’s intentions, knowledge, and considerations regarding rooming-in and breastfeeding and examined the influence of prenatal SDM on postpartum rooming-in and breastfeeding practices in Taiwan. Our results indicate a gap in women’s understanding of rooming-in compared with their knowledge of the benefits of breastfeeding. Before receiving prenatal education, the enrollees displayed more comprehensive knowledge about breastfeeding than about rooming, despite some uncertainty surrounding various aspects of breastfeeding, particularly the effects of mixed feeding on breast milk production. The main concerns influencing the intention to room in were rest, physical comfort, and the acquisition of skills for newborn care. Notably, only about 40% of women stated during prenatal SDM that they would prefer 24-h rooming-in. Factors such as older maternal age, full-time employment, having a graduate degree, and concerns about rest being disrupted negatively affected women’s prenatal intention to room in. Moreover, the implementation of prenatal SDM led to a declining trend in rooming-in during hospitalization, as well as EBF rates during the hospital stay, extending to 1 month and 2 months postpartum.

A possible explanation for the limited understanding of rooming-in compared with breastfeeding may be that approximately half of the women included in this study were experiencing their first pregnancy. Their lack of childbirth experience may have limited their ability to fully grasp what postpartum rooming-in entails. Moreover, for most Taiwanese women, access to information on rooming-in is limited unless they actively seek prenatal education. Despite the Taiwanese government’s efforts to promote prenatal education by subsidizing it during prenatal checkups, an online survey conducted in 2023 by the Birth Empowerment Alliance of Taiwan involving 2,157 pregnant women and their partners revealed that around 60% had not participated in any prenatal education [31]. Additionally, although the Taiwanese government provides a national hotline, official website, and app for information [4], informal sources (such as websites, friends, and family) may perpetuate stereotypes about rooming-in that influence maternity care decisions [32, 33].

One explanation for the confusion of the women in this study regarding the effects of mixed feeding on breast milk production may be the breastfeeding guidelines, which suggest that regular hand expression of milk can be used to maintain lactation when direct breastfeeding is not possible due to employment or other factors [34]. Additionally, the responses to the PDAs may reflect a preference for varied breastfeeding practices in their lives, contrasting with the standardized procedure taught in hospitals, which fails to offer individualized guidance [12, 35], highlighting a gap between women’s expectations and the advice they receive.

The women in this study were aware of the benefits of breastfeeding and the importance of rooming-in as well as the expectation of learning newborn care skills. Despite this, they emphasized the need for rest during the postpartum rooming-in period. This need is likely influenced by the traditional Taiwanese postpartum confinement practice known as “doing the month” [36], in which a woman is required to recuperate for 1 month following childbirth. Furthermore, our findings underscore the value of professional support during women’s hospital stays to fulfill their need for physical recovery and rest while also providing guidance and support to enhance their ability to care for themselves and their newborns. It would be beneficial to explore ways to optimize nurse-to-patient ratios in baby-friendly healthcare facilities to provide adequate support [37].

Women who chose partial rooming-in or separate care tended to be older, employed full-time, and hold graduate degrees. There is one possible explanation for this phenomenon. In Taiwan, invisible forms of gender inequality persist, with women expected to bear the primary responsibility for family care. Lida (2023) interviewed women between 30 and 40 years old with a bachelor’s degree or higher about their plans to balance family and career. The findings revealed that these women strongly resisted the traditional expectations of a “fixed life course.” These women perceived motherhood as unpaid and unequal, expressing dissatisfaction with policies intended to support families. Consequently, women who experience gender equality in public life may not wish to compromise their status by embracing motherhood [38]. Gender inequality and insufficient maternity protection policies are considerable structural barriers to creating a breastfeeding-friendly environment [39]. Additionally, perceptions of age-related risks may influence rooming-in decisions. Pregnant women of older age may feel a greater sense of risk and heightened anxiety regarding their pregnancies, leading to a belief that they or their baby may require special postnatal care and to doubts regarding their ability to care for themselves and their infant [40–42]. The experiences and perspectives of Taiwanese women regarding rooming-in and breastfeeding warrant further investigation.

### Study limitations

This is the first study in Taiwan to examine women’s intentions, knowledge, and considerations regarding rooming-in and breastfeeding during prenatal SDM and to explore the effects of implementing prenatal SDM on postpartum rooming-in and EBF. Nonetheless, this study has some limitations. First, the retrospective medical review design prevented the collection of data on some variables, which may have influenced the results. Second, the reliability and validity of the PDA used with the women included in this study remain uninvestigated. Third, this study’s representativeness is affected by our collection of data from only one BFHI-certified medical center in southern Taiwan. Integrating prenatal SDM data from other hospitals would be challenging because BFHI hospitals may tailor their PDA content for their patients, and accessing these data requires government authorization. Because of these limitations, our findings must be interpreted with caution.

## Conclusions

Rooming-in is considered crucial for parent–child bonding, learning infant cues, and the process of breastfeeding and care. Countries such as Taiwan, where a substantial number of births occur in BFHI-certified hospitals, should advocate for prenatal SDM. This process enables healthcare providers to identify and address potential misconceptions through personalized education while understanding women’s perceptions and unmet needs during hospitalization to enhance their intention to room in and breastfeed. Furthermore, it is necessary to establish comprehensive rooming-in care guidelines and to incorporate reasonable nurse-to-patient ratios into BFHI accreditation standards to ensure that women receive effective and sufficient professional support. These efforts will foster the cultivation of an environment that is baby- and mother-friendly.

## Data Availability

All datasets are available from the corresponding author upon reasonable request.

## Abbreviations

BFHI: Baby-Friendly Hospital Initiative
C-Section: Cesarean section
EBF: Exclusive breastfeeding
PDA: Patient decision aids
SDM: Shared decision-making
SD: Standard deviation
WHO: World Health Organization
VD: Vaginal delivery
